# PCB-Related Alteration of Thyroid Hormones and Thyroid Hormone Receptor Gene Expression in Free-Ranging Harbor Seals (*Phoca vitulina*)

**DOI:** 10.1289/ehp.8661

**Published:** 2006-03-16

**Authors:** Maki Tabuchi, Nik Veldhoen, Neil Dangerfield, Steven Jeffries, Caren C. Helbing, Peter S. Ross

**Affiliations:** 1 Institute of Ocean Sciences, Fisheries and Oceans Canada, Sidney, British Columbia, Canada; 2 Department of Biochemistry and Microbiology, University of Victoria, Victoria, British Columbia, Canada; 3 Washington Department of Fish and Wildlife, Tacoma, Washington, USA

**Keywords:** endocrine disruption, gene expression, marine mammal, PCBs, persistent organic pollutant, polychlorinated biphenyls, POP, seal, thyroid, thyroid hormone receptor

## Abstract

Persistent organic pollutants are environmental contaminants that, because of their lipophilic properties and long half-lives, bioaccumulate within aquatic food webs and often reach high concentrations in marine mammals, such as harbor seals (*Phoca vitulina*). Exposure to these contaminants has been associated with developmental abnormalities, immunotoxicity, and reproductive impairment in marine mammals and other high-trophic-level wildlife, mediated via a disruption of endocrine processes. The highly conserved thyroid hormones (THs) represent one vulnerable endocrine end point that is critical for metabolism, growth, and development in vertebrates. We characterized the relationship between contaminants and specific TH receptor (*TR* ) gene expression in skin/blubber biopsy samples, as well as serum THs, from free-ranging harbor seal pups (*n* = 39) in British Columbia, Canada, and Washington State, USA. We observed a contaminant-related increase in blubber *TR-*α gene expression [total polychlorinated biphenyls (∑PCBs); *r* = 0.679; *p* < 0.001] and a concomitant decrease in circulating total thyroxine concentrations (∑PCBs; *r* = −0.711; *p* < 0.001). Consistent with results observed in carefully controlled laboratory and captive feeding studies, our findings suggest that the TH system in harbor seals is highly sensitive to disruption by environmental contaminants. Such a disruption not only may lead to adverse effects on growth and development but also could have important ramifications for lipid metabolism and energetics in marine mammals.

A wide range of chemicals produced either directly or indirectly as a result of human activities have contributed to the contamination of aquatic food chains around the world. Marine mammals occupying high trophic levels in aquatic food webs are often contaminated with relatively high concentrations of persistent organic pollutants (POPs), including polychlorinated biphenyls (PCBs), polychlorinated dibenzo-*p*-dioxins (PCDDs), polychlorinated dibenzofurans (PCDFs), and polybrominated diphenylethers (PBDEs). This is because of food-web–related biomagnification, the extent of which reflects the persistence of the chemical coupled with the long lifespan and limited detoxification capacity of marine mammals (Ross et al. 2000; Tanabe et al. 1988). Many studies have shown that exposure to these complex mixtures of POPs can lead to developmental abnormalities, reproductive impairment, endocrine disruption, and immunosuppression in harbor seals (Brouwer et al. 1989; De Swart et al. 1994; Reijnders 1986) and other marine mammals (Skaare et al. 2001; Sonne et al. 2004). Marine mammals may therefore serve as indicators of marine environmental contamination, something that is relevant to both human and ecologic risk assessments (Ross 2000).

Harbor seals (*Phoca vitulina*) are non-migratory (adult home range of ~ 50 km^2^) and abundant around the coast of British Columbia, Canada, and Washington State, USA (Cottrell et al. 2002). The biology and physiology of this pinniped have been well documented, reflecting its wide distribution in temperate waters around the world and its relative ease of study. In British Columbia and Washington State, the harbor seal has been used to identify regional POP hotspots (Ross et al. 2004) and as a model marine mammal species for evaluating the relationship between contaminants and health effects (Levin et al. 2005; Simms et al. 2000).

Although the concept of endocrine disruption in wildlife has garnered much international scientific attention (Colborn et al. 1997), contaminant-related alteration of thyroid hormones (THs) and related processes may adversely affect vertebrates. The THs thyroxine (T_4_) and triiodothyronine (T_3_) play a crucial role in developmental processes and in the regulation of metabolism in adults. THs are produced in the thyroid gland, mainly in the form of T_4_, and are subsequently converted to the more bioactive T_3_ form in peripheral (target) tissues through the action of deiodinases. Laboratory-based studies have indicated that some POPs and their metabolites interfere with TH physiology at multiple levels, including hormone synthesis, circulatory transport of TH, and TH metabolism in the liver and brain (Brouwer et al. 1998; Legler and Brouwer 2003). Reductions in circulating TH levels have been observed with increasing exposure to PCBs and related compounds in laboratory animals, aquatic birds, and both captive and free-ranging marine mammals, highlighting the sensitivity of this endocrine end point to disruption by environmental contaminants (Rolland 2000).

In addition to effects on circulating THs, recent *in vitro* and laboratory animal evidence suggests that PCB and PCDD exposure can affect TH receptor (TR) activity and TH-responsive gene expression (Zoeller 2005). THs (primarily through the biologically active form of T_3_) function as signaling molecules that interact with two nuclear receptors, TR-α and TR-β [whose genes are designated as *THRA* and *THRB*, respectively, in GenBank (2006)], and alter their transcription activation and repression activities (Yen 2001). THs are therefore critical to the regulation of the gene expression machinery required during different life stages of an animal.

Although circulating TH levels are often used as biomarkers of contaminant exposure in wildlife (Chiba et al. 2001; Hall et al. 1998; Jenssen et al. 1995), gene expression end points that exploit the cellular functions of TH could provide a more sensitive and mechanistically based means to characterize the thyroid-toxic potential of complex contaminant mixtures in the real world. Such gene expression analysis might also form the basis of an early detection approach for POP exposure before the manifestation of higher-level health effects, such as developmental abnormalities and neurotoxicity, especially when results are consistent with laboratory-based observations.

Obtaining liver or blood from free-ranging marine mammals is generally fraught with logistical and ethical challenges. Skin/blubber biopsies have been used to generate useful information on contaminant concentrations and, more recently, on toxicologically relevant endocrine end points (Fossi et al. 2003; Miller et al. 2005; Mos and Ross 2002). Gene expression analysis using small biopsies has the potential to become a useful, sensitive, and minimally invasive biomarker of contaminant exposure in seals and other wildlife.

The objectives of this study were *a*) to develop *TR* gene expression biomarkers using skin/blubber biopsies, *b*) to confirm the utility of using circulating THs as biomarkers of POP exposure, and *c*) to assess the feasibility of using TH-related gene expression biomarkers in free-ranging harbor seals.

## Materials and Methods

### Sample collection

**Figure 1:**
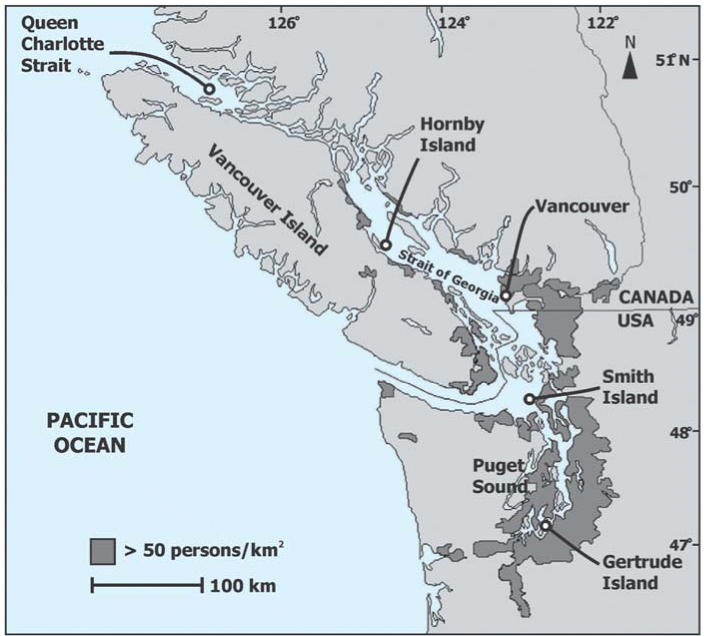
Harbor seal pups were live-captured from three sites (Queen Charlotte Strait, Hornby Island, and Vancouver) in British Columbia, Canada, and two sites (Smith Island in Juan de Fuca Strait and Gertrude Island in Puget Sound) in Washington State, USA. Human population densities > 50 persons/km^2^ are a proxy for possible regional contaminant sources. From State of Washington Office of Financial Management (2000) and Statistics Canada (2001).

A total of 39 healthy, young harbor seals (*Phoca vitulina*) of comparable body weight and condition were live-captured from five locations in southern British Columbia and northern Washington State during the summer of 2003 (Figure 1). These locations included three Canadian sites in Queen Charlotte Strait (QCS) (northeastern Vancouver Island, *n* = 10) and the Strait of Georgia (City of Vancouver, *n* = 8; Hornby Island, *n* = 7), and two U.S. sites in Juan de Fuca Strait (Smith Island, *n* = 7) and Puget Sound (Gertrude Island, *n* = 7). Both the accumulation of POPs and biologic end points in marine mammals are age dependent (Cottrell et al. 2002), so we restricted our sampling to pups ranging in age from 3.5 to 5 weeks. Seals hauled out on sandy beaches were captured using a rapidly deployed seine net (Jeffries et al. 1993), whereas those hauled out on rocky inlets were captured one at a time using a salmon-landing net (Simms and Ross 2000). Seals were kept in hoop nets until sampling and then removed from the net and manually restrained for tissue and blood collection. Seals were typically captured at low tides (peak haul-out times), with time of capture during the day ranging from 0825 hr to 1540 hr across all sites.

Blood samples were taken from the extradural vein using a Vacutainer blood collection system with an 18-gauge needle and serum collection tube (Becton-Dickenson, Franklin Lakes, NJ, USA). All collected blood samples were stored at 4°C in the field. Blood samples were centrifuged within 5 hr after collection at 400 × *g* for 20 min. Serum was aspirated and stored in 1 mL aliquots in cryovials either on dry ice (−80°C) or in liquid nitrogen (−196°C) during transport, and in a freezer (−80°C) until analysis of TH concentrations was performed.

Skin/blubber biopsy samples were taken from an area 20 cm lateral to the spinal column and anterior to the pelvis. The area was shaved first with an electric shaver (Sculptor with type-50 blades; Oster, Niles, IL, USA) and cleaned using Betadine (Purdue Frederick, Pickering, Ontario, Canada) followed by 95% isopropyl alcohol. Two biopsy samples were collected: one 3.5 mm in diameter and the other 8 mm, with each approximately 2–3 cm in depth (Acuderm, Ft. Lauderdale, FL, USA). After sample collection, the biopsy area on the animal was disinfected using Betadine and Aquaphor (Beiersdorf, Wilton, CT, USA) iodine ointment. The 8-mm-diameter biopsy samples were wrapped in hexane-rinsed aluminum foil, placed in 2 mL cryovials, and stored immediately in liquid nitrogen in the field. The 3.5-mm-diameter biopsy samples were placed into 1.0 mL of the RNA stabilization solution RNAlater (Ambion, Houston, TX, USA) in RNase-free 1.5 mL cryovials and stored on wet ice in the field. Blubber samples frozen in liquid nitrogen were subsequently transferred to −80°C storage in the laboratory, and biopsy samples in RNAlater were stored at −20°C.

Animals were subsequently weighed, sexed, measured for length and axillary girth, assessed for general body condition, and then released. Captive time was approximately 15–20 min for captures using the landing net and less than 60 min for captures using the seine net method. All procedures were carried out under the auspices of the respective animal care committees and scientific research permits for researchers in British Columbia [Fisheries and Oceans Canada Animal Care Committee using guidelines from the Canadian Council on Animal Care (CCAC 1997); Scientific Research Permit] and in Washington State under U.S. Marine Mammal Protection Act Scientific Research Permit No. 835 [National Oceanic and Atmospheric Administration (NOAA) 1997] issued to the Washington Department of Fish and Wildlife by National Marine Fisheries Service.

### Tissue homogenization

**Figure 2:**
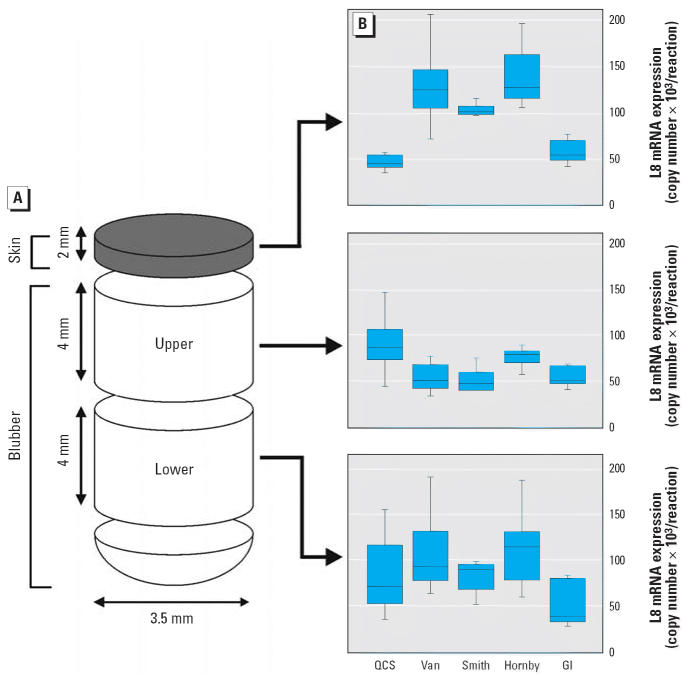
*L8* expression in different sections of harbor seal biopsy samples. Steady-state L8 mRNA levels were measured using total RNA isolated from skin and upper and lower sections of blubber. (*A*) Diagram of the specific tissue regions examined. (*B*) L8 mRNA expression levels for each tissue section indicated. Animals from five geographic locations were compared: QCS (*n* = 5), Vancouver (Van; *n* = 8), Smith Island (*n* = 7), Hornby Island (*n* = 7), and Gertrude Island (GI; *n* = 6). Each box plot shows the median, first and third quartiles, and maximum and minimum population values.

Because a possible stratification within blubber biopsies could influence our results, we evaluated the steady-state levels of the normalizer gene ribosomal protein L8 (*L8*) in skin and upper and lower blubber sections collected from all animals (Figure 2A). For this, each 3.5-mm-diameter tissue biopsy was separated into skin (~ 2 mm) and blubber sections using a razor blade before homogenization. Blubber samples were further divided into lower (close to the muscle) and upper (close to the skin) sections of 4 mm in depth.

All blubber samples were homogenized in TRIzol reagent (Invitrogen Canada Inc., Toronto, Ontario, Canada) using a Retsch MM301 mixer mill as described by Veldhoen and Helbing (2001) and with the following modifications. Each blubber tissue sample was homogenized in a 1.5 mL microcentrifuge tube with the addition of 400 μL TRIzol and a 3-mm-diameter tungsten-carbide bead. For any given sample, an additional 3–6 min of mixing was performed if unhomogenized tissue remained after the initial 6 min homogenization period. Because of the presence of a substantial amount of connective tissue, the mixer mill procedure was incapable of efficient homogenization of the skin samples. These samples were homogenized using a PowerGen 125 tissue homogenizer (Fisher Scientific, Pittsburgh, PA, USA). Skin samples were minced with a razor blade and placed into a 2.0 mL microcentrifuge tube (Mic Rew Simport Plastics Ltd., CA, USA) containing 400 μL TRIzol. The shearing head was placed directly into each sample tube and gradually ramped from 8,000 rpm to approximately 30,000 rpm for a total of 3 min with 10-sec cooling period intervals every 15 sec of homogenization. To minimize heat production in the skin samples, tubes were kept on wet ice during the entire homogenization procedure.

### Isolation of total RNA and preparation of cDNA

Total RNA was isolated from the tissue homogenates in TRIzol reagent as described by the manufacturer. After phase separation, 1 μL glycogen (Roche Diagnostics, Laval, QC, Canada) was added to each retained aqueous phase, and RNA was precipitated with the addition of isopropanol and a 1 hr incubation at −20°C. Total RNA was resuspended in diethyl pyrocarbonate–treated distilled, deionized H_2_O (20 μL for blubber and 40 μL for skin samples) and stored at −70°C.

Total cDNA was produced using Superscript II RNase H^−^ reverse transcriptase as described by the manufacturer (Invitrogen Canada Inc.). One microgram of total RNA from each sample was annealed with 500 ng random hexamer oligonucleotide (Amersham Biosciences Inc., Baie D’urfe, Quebec, Canada) at 65°C for 10 min and placed on wet ice. The assembled 20 μL cDNA synthesis reactions were incubated at 42°C for 2 hr and diluted 20-fold before real-time quantitative polymerase chain reaction (QPCR) analysis.

### Cloning of TR cDNA sequences

Target cDNA sequences representative of the gene transcripts *TR-*α and *TR-*β as well as our control gene, *L8*, were amplified using primers designed using Primer Premier software (version 4.1; Premier Biosoft International, Palo Alto, CA, USA) and synthesized by AlphaDNA (Montreal, Quebec, Canada) (Table 1). Each 25 μL DNA amplification reaction included 2 μL of 20-fold total cDNA template, 20 pmol of each primer, 200 μM equimolar dNTPs (dATP, dCTP, dGTP, and dTTP), and 2.5 units of Taq DNA polymerase (Invitrogen Canada Inc.). DNA amplification was performed on a Gene Amp PCR System 9700 (PerkinElmer Biosystems, Foster City, CA, USA) using the following thermocycle conditions: denaturation at 95°C (5 min); 35 cycles of 94°C (1 min), 53°C (1 min), and 72°C (2 min); and an elongation step at 72°C (7 min). DNA products were separated on a 1.5% agarose gel and visualized with ethidium bromide staining on a ChemiImager 4000 (Alpha Innotech Corp., San Leandro, CA, USA). DNA bands representing *TR-*α [631 base pairs (bp)], *TR-*β (801 bp), and *L8* (602 bp and 126 bp) were excised and isolated by three repeated 5-min freeze/thaw cycles followed by a 10 min centrifugation at 12,000 × *g* (Smith 1980). Isolated cDNA products (4 μL) were cloned into pCR2.1-TOPO vector using the TOPO TA Cloning Kit (Invitrogen Canada Inc.). Plasmid DNA was purified from selected transformants using the QIAprep Spin Miniprep Kit (Qiagen, Mississauga, Ontario, Canada), and the presence of insert sequence was confirmed by restriction analysis using Eco*RI* (Amersham Biosciences). The identity of each cloned cDNA was determined by DNA sequencing followed by BLASTn analysis (National Center for Biotechnology Information 2006). Primer sequences are shown in Table 1, and cloned sequences have been submitted to GenBank (2006).

### QPCR assay

Primers specific for seal *TR-*α, *TR-*β, and *L8* were designed for the reverse-transcription QPCR assay (Table 1). Quantitative DNA amplification reactions (15 μL) were performed on a MX4000 system (Stratagene, La Jolla, CA, USA) as described previously (Crump et al. 2002). Each sample was prepared in quadruplicate, and the derived copy number values were averaged. The copy number for each gene transcript was determined from standard curves generated from the cloned plasmids in the previous section. *TR-*α and *TR-*β expression values were normalized to those of the expression of the *L8* internal control. The expression of this gene has been found to be invariant in many tissue types during developmentally associated changes in endogenous TH concentrations in reptiles (Katsu et al. 2004) and amphibians (Shi and Liang 1994).

### TH assay

The concentrations of total T_4_ (TT_4_), free T_4_ (FT_4_), total T_3_ (TT_3_), and free T_3_ (FT_3_) were measured in animals from all five locations (*n* = 39) using related EIAgen enzyme-linked immunosorbent assay (ELISA) kits and by following the manufacturer’s recommended protocol (Adaltus, Montréal, Quebec, Canada). Frozen (undiluted) serum samples were thawed on wet ice, and all four TH measurements were obtained within 6 hr. For each ELISA assay, reactions were prepared in triplicate, and the signal intensity of seal serum samples and TH standards was measured at 450 nm on an MRX microplate reader (Dynatech Laboratories Inc., Chantilly, VA, USA). The sample data were subsequently averaged and compared with the standard curve in order to obtain representative TH concentration values.

Interassay variation was evaluated in two ways. First, we regularly included a pooled seal serum sample as a reference, and results were accepted for any given assay only when reference results were ± 20% of expected values. Second, total hormone measurements (TT_3_ and TT_4_) were validated using the manufacturer’s reference standard (Thyroid Calver reagent; Casco Neal, Portland, ME, USA), and results were accepted for an assay only when concentrations were within ± 5% of expected values.

No purified harbor seal THs are commercially available. With this in mind, we validated the TH assays for harbor seals by conducting analyses of serial dilutions within a fixed sample volume and using incremental spikes of seal serum with Thyroid Calver reagent. Responses of serial dilutions of seal serum and standard additions of seal serum with the reference standard both produced linear results (data not shown).

### Measurement of POP concentrations in blubber tissue

Each frozen (−80°C) 8 mm tissue biopsy was cut vertically, and the upper skin layer (~ 2 mm) removed. A portion of each blubber sample (100–300 mg wet weight) was used in the analysis for all PCB congeners and for specific PCDD and PCDF congeners using high-resolution gas chromatography and high-resolution mass spectrometry analysis at the Fisheries and Oceans Canada Regional Contaminant Laboratory (Institute of Ocean Sciences, Sidney, British Columbia, Canada). Details of the chromatography and mass spectrometry conditions, the criteria used for chemical identification and quantification, and the quality assurance and quality control practices have been previously described (Ikonomou et al. 2001).

Although 154 PCB peaks were quantified (out of 209 theoretical congeners), many congeners were not detectable in all of the samples. ∑PCB concentration was therefore calculated using the following rules. If a congener was detected in > 70% of the sample population, the minimum detection limit substitutions were made. Where congeners were detected in < 70% of samples, the minimum detection limit was set at zero. Sample lipid values were also measured, and the concentration of POPs was expressed on a lipid weight (lw) basis. Total toxic equivalents (∑TEQs) to 2,3,7,8-tetrachlorodibenzo-*p*-dioxin were calculated for all dioxin-like PCBs (12 congeners), PCDD (7 congeners), and PCDF (10 congeners) using the most recently reported international mammalian toxic equivalency factors (Van den Berg et al. 1998).

### Statistical analyses

All statistical analyses were performed using SPSS software (version 12; SPSS Inc., Chicago, IL, USA). Results were evaluated by site and among all individuals. For the former, harbor seal pups from five sites were compared for circulating TH concentrations and steady-state mRNA expression levels in skin and blubber biopsy samples. Seals from the remote QCS, previously (and in this study) shown to be relatively uncontaminated (Ross et al. 2004), were used as a reference group. For each group, values were examined for normality with the Shapiro-Wilk test and for homogeneity of variances using Levine’s test. Groups that were normal with equal variance were evaluated using one-way analysis of variance (ANOVA) to assess intergroup differences followed by Tukey’s honest significant difference (HSD) test. If the data were not normally distributed, a Kruskal-Wallis test was used followed by a Mann-Whitney *U* test for pairwise comparison of groups. Significance was defined as *p* < 0.05. Extreme outliers, defined as values more than three times the interquartile range, were removed.

For the among-individual assessment of the entire study group, correlation analysis was carried out using the Pearson method for normally distributed data or the nonparametric Kendall’s tau-b method when data were not normally distributed. Given our concern that body weight (~ age) of the harbor seal pups might influence either contaminant concentrations or thyroid end points, we conducted regressions between body weight and contaminant concentrations, TH concentrations, and TR levels. If body weight exhibited a significant relationship with contaminant or thyroid measurements, we conducted multiple regression analysis to identify the relative contribution of each variable.

## Results

### Sample collection

Of the 39 harbor seals sampled, availability of adequate tissue quality and cost considerations for contaminant analyses resulted in the analysis of 39 serum samples for TH measurement, 35 biopsies (3.5 mm) for gene expression analysis, and 24 blubber biopsies (8 mm) for contaminant analysis.

The mean ± SE body weight was 20.6 ± 0.52 kg (range, 14.1–27.0 kg). Our ANOVA results revealed a significant difference among sites. A subsequent Tukey’s HSD test indicated that only Smith Island seals differed, being slightly heavier than QCS seals (*p* = 0.038).

### TR *gene sequences in the harbor seal*

Partial cDNA sequences were isolated from biopsied harbor seal blubber that represented expressed mRNA of *TR-*α and *TR-*β genes, as well as our control gene, *L8*. Both *TR* sequences obtained are predicted to encompass approximately half of the estimated open reading frame region within the mRNA transcripts and include sequence located between the encoded DNA-binding and ligand-binding domains. A comparison of harbor seal *TR* sequences with six other species using the ClustalW alignment program (European Bioinformatics Institute 2006) indicated that the mRNA sequence for *TR-*α and *TR-*β are highly conserved among these vertebrates (Table 2). This is particularly evident within mammals, where the putative protein sequences of harbor seal *TR-*α and *TR-*β display > 99% amino acid identity.

### *Measurement of* TR *expression in skin/blubber biopsies*

Based on the cDNA sequence obtained, oligonucleotide primers were developed for QPCR analysis of specific gene expression biomarkers. Significant variation in L8 mRNA expression (*p* < 0.05, Tukey’s HSD or Mann-Whitney *U*-test) within the vertical plane of the biopsy tissue sample was observed for all sample sections, with the exception of QCS and Gertrude Island (Figure 2B). We then compared each section (skin, upper blubber, and lower blubber) separately across the animals from different locations. Both the skin and the upper blubber (adjacent to skin) sections showed a significant difference in L8 mRNA expression among sites (skin: *p* < 0.001, Kruskal-Wallis; upper blubber: *p* = 0.028, Kruskal-Wallis). However, *L8* steady-state transcript levels in the lower blubber region did not differ among sites (*p* = 0.05, ANOVA; *p* > 0.05, Tukey). Therefore, we chose the amount of *L8* transcript in the lower blubber as a suitable normalizer gene for the comparison of gene expression levels between the different seal populations. All subsequent QPCR analyses of *TR* transcript copy numbers are presented for lower blubber sections only.

TR-α mRNA abundance was found to be significantly higher than that of TR-β (*p* = 0.004, Tukey) in all the individuals examined (Table 3). In addition, the relationship between TR-α and TR-β mRNA expression patterns was positively correlated (*R* = 0.651). In comparisons of animals from different geographic locations, Gertrude Island samples displayed a significant elevation in both *TR-*α (*p* < 0.001, Tukey) and *TR-*β (*p* = 0.011, Tukey) transcript levels compared with animals from the QCS reference site.

### Serum TH levels

The concentrations of different TH forms were measured in serum collected from the seal pups by site (Table 4). Among the seals sampled from different locations, Gertrude Island animals had significantly lower TT_4_ and FT_4_ compared with reference site QCS animals (*p* < 0.001, Tukey; *p* < 0.001, Mann-Whitney). A strong positive correlation was found between measured TT_4_ and FT_4_ levels (*R* = 0.844, *p* < 0.001) among all individuals, whereas no correlation existed between TT_3_ and FT_3_ serum concentrations (*R* = 0.260, *p* = 0.121). Negative correlations between circulating TT_4_ and TR-α mRNA levels (*R* = −0.456, *p* < 0.05) and circulating FT_4_ and *TR-*α expression (*R* = −0.481, *p* < 0.05) were detected. No correlation was observed between any serum TH measurement and *TR-*β transcript levels (data not shown).

### POP concentrations in blubber

Of a total of 154 PCB congener peaks quantified, 135 peaks were detected in QCS seals, 142 peaks in Smith Island seals, and 153 peaks in Gertrude Island seals. Four of 24 seals were identified as extreme outliers in the TEQ calculations and were removed from further analysis. Seal pups located on Gertrude Island showed an approximate 10-fold higher ∑PCB concentration (6.2 ± 1.0 mg/kg, lw) compared with animals from the reference QCS (0.7 ± 0.1 mg/kg, lw; *p* < 0.001, Tukey) and 5-fold higher than animals from Smith Island (1.3 ± 0.2 mg/kg, lw; *p* < 0.001, Tukey). Calculated ∑TEQ values for PCBs, PCDDs, and PCDFs were also significantly higher in seal pups on Gertrude Island (70.4 ± 16.5 ng/kg) compared with animals from QCS (12.6 ± 2.2 ng/kg; *p* < 0.001, Mann-Whitney) but did not differ significantly from those from Smith Island (24.9 ± 11.6 ng/kg; *p* = 0.052, Mann-Whitney). PCBs were the major constituent measured among contaminant classes measured in study animals, which included PCBs, PCDDs, and PCDFs, and were also the dominant contributor to ∑TEQ (PCBs represented an average of 43.4% in QCS seals, 59.2% for Smith Island seals, and 90.1% for Gertrude Island seals). More details on contaminant levels and patterns in harbor seals from this region are available elsewhere (Ross et al. 2004).

### *Correlation of TH and* TR *end points with POP exposure*

**Figure 3:**
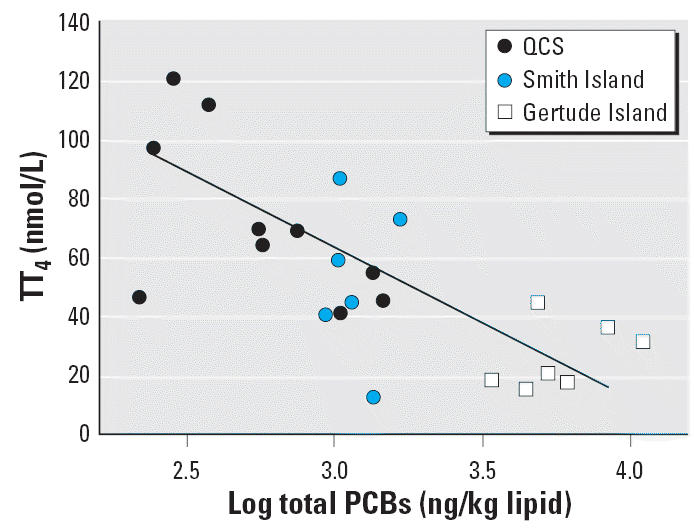
Correlation analysis of circulating TT_4_ levels with ∑PCB measured in blubber of harbor seal pups from the southern coast of British Columbia and northern Washington State. A significant negative correlation is noted. *R* = −0.711; *p* < 0.01.

**Figure 4:**
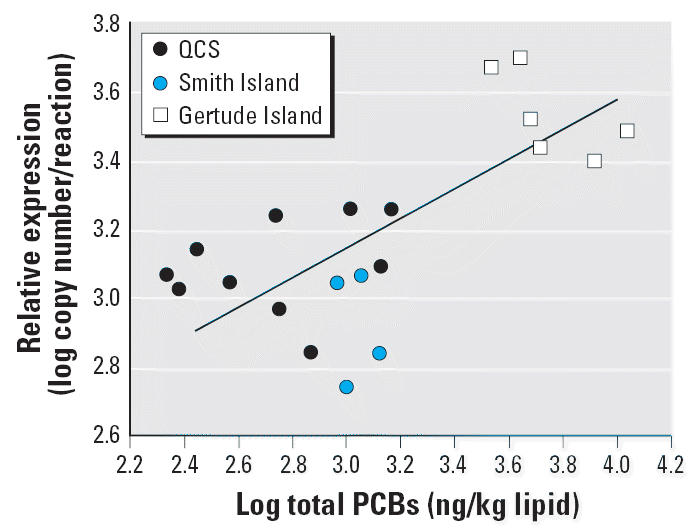
Correlation analysis of relative TR-α mRNA expression with ∑PCB concentrations measured in the lower blubber biopsy section of harbor seal pups from the southern coast of British Columbia and northern Washington State. A significant positive correlation is noted. *R* = 0.679; *p* < 0.01.

∑PCB concentrations were negatively correlated with circulating TT_4_ (*R* = −0.711, *p* < 0.001) (Table 5, Figure 3) and FT_4_ (*R* = −0.724, *p* < 0.001, Table 5). In contrast, a positive correlation was observed between ∑PCB concentrations and the level of TR-α mRNA (*R* = 0.679, *p* < 0.01) (Table 5, Figure 4). Similarly, negative correlations were also found between ∑TEQ and circulating TT_4_ (*R* = −0.495, *p* < 0.01), and positive correlations with the level of *TR-*α expression in the blubber (*R* = 0.464, *p* < 0.01).

Although we limited our studies to seals of a similar body weight (~ age), the potential confounding influence of age on both PCB concentration and thyroid end points remained a concern. Subsequent regression analysis revealed that body weight did not correlate with TT_3_, *TR-*α, *TR-*β, ∑PCBs, or ∑TEQ (data not shown). However, there were negative correlations between body weight and TT_4_, FT_3_, and FT_4_. We found no correlation between FT_3_ and PCBs, so we did not further evaluate this relationship. Multiple regression analysis showed that, although both PCB concentrations and body weight correlated with TT_4_ and FT_4_, PCBs were the primary exploratory variable in the observed thyroid changes, whereas body weight was not significant for TT_4_ (PCBs: partial *R* = 0.71, *p* < 0.001; body weight: partial *R* = 0.27, *p* = 0.08) or FT_4_ (PCBs: partial *R* = 0.72, *p* < 0.001; body weight: partial *R* = 0.33, *p* = 0.14). There were no significant correlations between any of the endocrine end points and time of day for each capture (data not shown), suggesting that circadian rhythm did not unduly influence our results. Likewise, there was no correlation between time held (restraint) before release and any of the endocrine end points, suggesting that stress was not a factor (data not shown).

## Discussion

A “weight of evidence” from laboratory-based studies, captive feeding studies of seals, and studies of free-ranging marine mammals highlights the endocrine-disrupting nature of complex mixtures of POPs and many of their constituents (Ross 2000). Although mechanisms of action are often ill-defined in field studies, a common pattern of adverse health effects observed in contaminant-exposed individuals and populations includes developmental, immunologic, and reproductive effects. Despite having been banned in North America for three decades, the highly persistent PCBs continue to present a toxic risk to wildlife and dominated the contaminant profiles in our study of British Columbia and Washington State harbor seals. Gertrude Island (Puget Sound) harbor seals were particularly contaminated, having PCB concentrations that were several times higher than those sampled in the adjacent coastal waters of northern Washington State and southern and central British Columbia.

Elevated POP exposure has been associated with altered circulating vitamin A and immune function in free-ranging harbor seals sampled from the same study areas (Levin et al. 2005; Simms et al. 2000). Our observed negative relationship between circulating TT_4_ and PCB concentrations in harbor seals contributes to the notion that PCBs represent a significant health concern at the top of the food chain. This finding is consistent with previous observations of contaminant-related reductions in TH concentrations in captive seals fed contaminated fish (Brouwer et al. 1989) and in free-ranging pinnipeds (Chiba et al. 2001; Debier et al. 2005; Hall et al. 1998; Jenssen et al. 1995). Histopathologic lesions, including fibrosis and colloid depletion, in thyroid glands of seals inhabiting PCB-contaminated areas (Schumacher et al. 1993) may explain reduced TH levels in our contaminated seals. However, laboratory animal studies provide more information on possible mechanisms of action, where altered hormone synthesis in the thyroid gland, disrupted circulatory transport, and altered metabolic enzyme activity have been observed (Brouwer et al. 1998). Our findings suggest that the more contaminated seals from Gertrude Island may be considered hypothyroid (Haulena et al. 1998), highlighting concerns about the health of high trophic level wildlife in this region.

THs play a critical role in regulating a wide range of physiologic processes such as growth, development, and metabolism, largely through binding to the nuclear receptors *TR-*α and *TR-*β, and modulate their activity on TH-responsive gene promoters (Wu and Koenig 2000). PCBs can also directly affect TR activity and TH-responsive gene expression. The observed differential relationship between *TR-*α and *TR-*β transcripts in seal blubber samples relative to PCB levels may indicate a particular vulnerability of the *TR-*α gene. This may be related to the apparent hypothyroidism observed in the more contaminated animals. Interestingly, increased *TR-*α expression has been observed in the brains of hypothyroid compared with euthyroid rats (Constantinou et al. 2005). Positively TH-regulated genes were up-regulated in postnatal and fetal rats brains after *in utero* exposure to the PCB mixture Aroclor 1254 despite a reduction in the dam’s circulating TH levels (Gauger et al. 2004; Zoeller et al. 2000). These results suggest that PCBs may interfere directly or indirectly with TH signaling, leading to changes in TH-responsive gene expression.

Altered circulating TH levels in PCB-exposed marine mammals provide evidence of an effect on this endocrine end point. However, obtaining blood samples is not always feasible, and skin/blubber biopsies essentially represent the only obtainable samples for many marine mammals, including cetaceans. In addition, circulating TH levels can be influenced by a number of natural factors and may not present a rigorous assessment of thyroid status. We therefore developed a gene expression biomarker approach using *TR* expression levels in blubber/skin biopsies in order to evaluate the utility of such an approach for harbor seals and other marine mammals. Using this biopsy-based sampling technique, we were able to quantify the expression of *TR* genes in blubber. Although we focused on *TR* expression in these studies, other emerging gene expression biomarkers could be examined in the same way. Expression levels of the aryl hydrocarbon receptor or cytochrome P450 as gene expression biomarkers in liver have already been suggested for marine mammals (Kim and Hahn 2002).

The positive correlation between blubber *TR-*α and PCB concentrations in harbor seals suggests that contaminants either directly or indirectly affect this TH end point and may alter TH-regulated gene expression. The high degree of sequence conservation between harbor seals and other vertebrates accentuates the likely functional similarity of TRs between animal groups. Although our results would suggest that PCBs affect systemic thyroid homeostasis in harbor seals, our detection of contaminant-related alteration of *TR* gene expression in blubber raises a toxicologic concern of particular note for marine mammals. TH is known to play an important role in the maintenance and function of adipose tissue (Ailhaud et al. 1992). T_3_ treatment can induce adipocyte cell proliferation, fat cell cluster formation, lipid accumulation, and increased malic enzyme and glycerophosphate dehydrogenase activities in young rats as well as in preadipocyte cell lines (Flores-Delgado et al. 1987; Grimaldi et al. 1982). TRs within murine adipocytes predominantly are composed of the *TR-*α isoform, with little detectable *TR-*β isoform (Jiang et al. 2004), consistent with our findings in blubber. Recently, several TH-regulated genes were identified in human and mouse adipose tissue that encode for protein products involved in lipid metabolism (Viguerie et al. 2002).

Blubber is a specialized adipose tissue layer under the skin of marine mammals and is vital for energy storage, heat insulation, thermogenesis, and buoyancy control. Blubber is typically viewed as a storage depot associated with lipid reserves, within which lipids, lipid classes, and fatty acid profiles have been characterized in physiologic and energetic studies of marine mammals (Iverson et al. 1997; Mellish et al. 1999). However, blubber also represents an important storage site for micronutrients, holding as much as 66% of the body burden of vitamin A in harbor seals (Mos and Ross 2002). Our finding of *TR-*α in blubber highlights the metabolically dynamic nature of this tissue because this receptor mediates the actions of TH-dependent metabolism and homeostasis. We speculate that contaminants might therefore present a risk to the structural and functional integrity of blubber because metabolism within adipocytes may be altered. The influence of TH-related processes on body weight in laboratory animals and in humans (Baxter et al. 2004; Pelletier et al. 2003) underscores the potential effects of a disruption of TR on such critical processes as energy storage and thermoregulation in marine mammals.

Our biomarker-based thyroid assessment may be applied to studies of other species for which blood samples are not available because of logistical constraints (e.g., cetaceans). The contaminant-associated decrease in circulating TH levels and concomitant up-regulation of *TR-*α expression in blubber of harbor seals may indicate an increased risk of TH-dependent health effects, such as developmental abnormalities and neurotoxicity. In addition, altered *TR-*α gene expression in blubber may have profound consequences for metabolic turnover and energetics in contaminated marine mammal populations.

## Figures and Tables

**Table 1:** DNA primers used in the cloning and QPCR analysis of harbor seal target genes.

Method	Gene	Primer name	GenBank accession no.	Amplicon size (bp)	Primer sequences
Cloning	Ribosomal protein *L8*^a^	UL8up	DQ212693	126	5′-GGTGTGGCTATGAATCCTGT-3′
		L8–2dn			5′-ACGACGAGCAGCAATAAGAC-3′
	Ribosomal protein *L8*	PV3	DQ212694	602	5′-CCGCCATGGGCCGTGTGATC-3′
		PV4			5′-CGTACTCGTGGCCAGCAGTT-3′
	*TR-*α	PV6	DQ212695	631	5′-TGCTGCATTATCGACAAGATCAC-3′
		PV8			5′-GTGACTTGCCCAGTTCAAAGATGG-3′
	*TR-*β	PV12	DQ212696	801	5′-TATTCCTGTAAATATGAAGG-3′
		PV16			5′-GTAATTGATATAGTGTTCAAA-3′
QPCR	*TR-*α	PV19	—	231	5′-CGACGGAAGGAGGAAATG-3′
		PV20			5′-GATCTTGGTAAACTCGCTGAA-3′
	*TR-*β	PV30	—	425	5′-AGAGGCTGGCAAAGAGGA-3′
		PV31			5′-ACTTTCTGGGTCATAGCG-3′

a These primers were also used for QPCR analysis.

**Table 2:** Comparison of harbor seal *TR-*α and *TR-*β cDNA and putative protein sequences of other species.

	*Phoca vitulina TR-*α			*Phoca vitulina TR-*β		
Species	GenBank accession no.	Nucleotide^a^ (584 bp)	Amino acid^a^ (194 aa)	GenBank accession no.	Nucleotide^a^ (760 bp)	Amino acid^a^ (253 aa)
*Homo sapiens*	BC035137	95	100	NM00461	94	100
*Ovis aries*	Z68308	96	100	Z68307	89	99
*Mus musculus*	BC046795	92	100	NM009380	90	99
*Gallus gallus*	NM205313	84	93	NM205447	86	95
*Danio rerio*	U54796	77	85	NM131340	75	89
*Xenopus laevis*	L76285	79	92	M35361	79	93

a Values represent identity for the comparable nucleotide and putative amino acid sequences.

**Table 3:** Steady-state levels of *TR-*α and *TR-*β transcripts measured in the lower blubber biopsy section from harbor seal pups in coastal British Columbia and Washington State.

	QCS (*n* = 10)	Vancouver (*n* = 7)	Hornby Island (*n* = 7)	Smith Island (*n* = 5)	Gertrude Island (*n* = 6)
*TR-*α	1,309 ± 125 (699–1,863)	1,341 ± 193 (590–1,927)	2,459 ± 531 (988–4,561)	802 ± 148 (464–1,185)	3,633 ± 446* (2,539–5,163)
*TR-*β	973 ± 246 (437–2,984)	799 ± 88 (519–1,254)	920 ± 194 (362–1,732)	511 ± 26 (458–603)	1,387 ± 142* (936–1,863)

Data are mean ± SE of the copy numbers per reaction (range). Data from QCS animals were used as the reference values for statistical analyses.

* Significant difference in gene expression (*p* < 0.05).

**Table 4:** Circulating TH concentrations in harbor seal pups from five sites in coastal British Columbia and Washington State.

	QCS (*n* = 10)	Vancouver (*n* = 8)	Hornby Island (*n* = 7)	Smith Island (*n* = 7)	Gertrude Island (*n* = 7)
TT_4_ (nmol/L)	71.8 ± 9.0 (41.1–120.5)	74.4 ± 6.6 (47.2–98.5)	59.4 ± 4.9 (43.3–81.5)	56.5 ± 9.8 (12.6–86.7)	26.3 ± 4.3* (15.1–44.8)
FT_4_ (pmol/L)	37.8 ± 2.4 (28.3– 49.5)	39.2 ± 2.6 (25.1–47.4)	32.7 ± 0.8 (29.2–35.5)	32.9 ± 3.0 (24.0–46.6)	22.3 ± 2.1* (16.9–32.7)
TT_3_ (nmol/L)	0.72 ± 0.14 (0.22–1.57)	0.80 ± 0.08 (0.55–1.18)	0.28 ± 0.08 (0.08–0.64)	0.64 ± 0.07 (0.28–0.84)	0.70 ± 0.10 (0.40–1.10)
FT_3_ (pmol/L)	7.61 ± 0.67 (4.49–10.15)	7.15 ± 0.51 (5.41–9.42)	7.91 ± 1.58 (3.69–12.13)	6.81 ± 0.58 (4.91–8.90)	8.99 ± 0.99 (5.17–13.33)

Data are mean ± SE (range). Data from QCS animals were used as reference values for statistical analyses.

* Significant difference in gene expression (*p* < 0.05).

**Table 5:** Correlation between contaminant concentrations (∑PCBs and ∑TEQ) in seal blubber and circulating TH concentrations and *TR* gene expression from the lower blubber biopsy section for all seals from five sites in British Columbia and Washington State.

	Serum TH				Blubber	
	TT_4_	FT_4_	TT_3_	FT_3_	*TR-*α	*TR-*β
∑PCBs	−0.711** (< 0.001)	−0.724** (< 0.001)	−0.111 (0.616)	0.108 (0.624)	0.679** (0.001)	0.360 (0.119)
∑TEQ	−0.495** (0.002)	−0.360* (0.027)	−0.042 (0.795)	0.053 (0.746)	0.464** (0.007)	0.373* (0.031)

Data are correlation coefficients (*p*-values).

* *p* < 0.05.

** *p* < 0.01.

## References

[b1-ehp0114-001024] Ailhaud G, Grimaldi P, Negrel R (1992). Cellular and molecular aspects of adipose tissue development. Annu Rev Nutr.

[b2-ehp0114-001024] Baxter JD, Webb P, Grover G, Scanlan TS (2004). Selective activation of thyroid hormone signaling pathways by GC-1: a new
approach to controlling cholesterol and body weight. Trends Endocrino Metab.

[b3-ehp0114-001024] Brouwer A, Morse DC, Lans MC, Schuur AG, Murk AJ, Klasson WE (1998). Interactions of persistent environmental organohalogens with the thyroid
hormone system: mechanisms and possible consequences for animal and
human health. Toxicol Ind Health.

[b4-ehp0114-001024] Brouwer A, Reijnders PJH, Koeman JH (1989). Polychlorinated biphenyl (PCB)-contaminated fish induces vitamin A and
thyroid hormone deficiency in the common seal (*Phoca vitulina*). Aquat Toxicol.

[b5-ehp0114-001024] CCAC (Canadian Council on Animal Care) 1997. CCAC Guidelines on Animal Use Protocol Review (1997). Available: http://www.ccac.ca/en/CCAC_Programs/Guidelines_Policies/GDLINES/PROTOCOL/PROTGDE.HTM [accessed 27 April 2006].

[b6-ehp0114-001024] Chiba I, Sakakibara A, Goto Y, Isono T, Yamamoto Y, Iwata H (2001). Negative correlation between plasma thyroid hormone levels and chlorinated
hydrocarbon levels accumulated in seals from the coast of Hokkaido, Japan. Environ Toxicol Chem.

[b7-ehp0114-001024] Colborn T Dumanoski D Myers JP 1997. Our Stolen Future. New York:Plume Printing.

[b8-ehp0114-001024] Constantinou C, Margarity M, Valcana T (2005). Region-specific effects of hypothyroidism on the relative expression of
thyroid hormone receptors in adult rat brain. Mol Cell Biochem.

[b9-ehp0114-001024] Cottrell PE, Jeffries SJ, Beck B, Ross PS (2002). Growth and development in free-ranging harbour seal (*Phoca vitulina*) pups from southern British Columbia. Mar Mamm Sci.

[b10-ehp0114-001024] Crump D, Werry K, Veldhoen N, Van Aggelen G, Helbing CC (2002). Exposure to the herbicide Acetochlor alters thyroid hormone-dependent gene
expression and metamorphosis in *Xenopus laevis*. Environ Health Perspect.

[b11-ehp0114-001024] Debier C, Ylitalo GM, Weise M, Gulland F, Costa DP, LeBoeuf BJ (2005). PCBs and DDT in the serum of juvenile California sea lions: associations
with vitamins A and E and thyroid hormones. Environ Pollut.

[b12-ehp0114-001024] De Swart RL, Ross PS, Vedder LJ, Timmerman HH, Heisterkamp SH, Van Loveren H (1994). Impairment of immune function in harbor seals (*Phoca vitulina*) feeding on fish from polluted waters. Ambio.

[b13-ehp0114-001024] European Bioinformatics Institute 2006. ClustalW Submission Form. Available: http://www.ebi.ac.uk/clustalw/2006 [accessed 27 April 2006].

[b14-ehp0114-001024] Flores-Delgado G, Marsch-Moreno M, Kuri-Harchch W (1987). Thyroid hormone stimulates adipocyte differentiation of 3T3 cells. Mol Cell Biochem.

[b15-ehp0114-001024] Fossi MC, Marsili L, Neri G, Natoli A, Politi E, Panigada S (2003). The use of a non-lethal tool for evaluating toxicological hazard of organochlorine
contaminants in Mediterranean cetaceans: new data 10 years
after the first paper published in MPB. Mar Pollut Bull.

[b16-ehp0114-001024] Gauger KJ, Kato Y, Haraguchi K, Lehmler H-J, Robertson LW, Bansal R (2004). Polychlorinated biphenyls (PCBs) exert thyroid hormone-like effects in
the fetal rat brain but do not bind to thyroid hormone receptors. Environ Health Perspect.

[b17-ehp0114-001024] GenBank 2006. GenBank Overview. Available: http://www.ncbi.nlm.nih.gov/Genbank/index.html [accessed 27 April 2006].

[b18-ehp0114-001024] Grimaldi P, Djian P, Negrel R, Ailhaud G (1982). Differentiation of Ob17 preadipocytes to adipocytes: requirement of adipose
conversion factor(s) for fat cell cluster formation. EMBO J.

[b19-ehp0114-001024] Hall AJ, Green NJL, Jones KC, Pomeroy PP, Harwood J (1998). Thyroid hormones as biomarkers in grey seals. Mar Pollut Bull.

[b20-ehp0114-001024] Haulena M, St Aubin DJ, Duignan PJ (1998). Thyroid hormone dynamics during the nursing period in harbour seals, *Phoca vitulina*. Can J Zool.

[b21-ehp0114-001024] Ikonomou MG, Fraser T, Crewe N, Fischer MB, Rogers IH, He T (2001). A comprehensive multiresidue ultra-trace analytical method, based on HRGC/HRMS, for
the determination of PCDDs, PCDFs, PCBs, PBDEs, PCDEs, and
organochlorine pesticides in six different environmental matrices. Can Tech Rep Fish Aquat Sci.

[b22-ehp0114-001024] Iverson SJ, Frost KJ, Lowry LF (1997). Fatty acid signatures reveal fine scale structure of foraging distribution
of harbor seals and their prey in Prince William Sound, Alaska. Mar Ecol Prog Ser.

[b23-ehp0114-001024] Jeffries SJ, Brown RF, Harvey JT (1993). Techniques for capturing, handling and marking harbour seals. Aquat Mamm.

[b24-ehp0114-001024] Jenssen BM Skaare JU Woldstad S Nastad AT Haugen O Kloven B et al. 1995. Biomarkers in blood to assess effects of polychlorinated biphenyls in free-living grey seal pups. In: Whales, Seals, Fish and Man (Blix AS, Walløe L, Ulltang Ø, eds). Amsterdam:Elsevier Science BV, 607–615.

[b25-ehp0114-001024] Jiang W, Miyamoto T, Kakizawa T, Sakuma T, Nishio S, Takeda T (2004). Expression of thyroid hormone receptor alpha in 3T3-L1 adipocytes; triiodothyronine
increases the expression of lipogenic enzyme and triglyceride
accumulation. J Endocrinol.

[b26-ehp0114-001024] Katsu Y, Bermudez DS, Braun EL, Helbing CC, Miyagawa S, Gunderson MP (2004). Molecular cloning of the estrogen and progesterone receptors of the American
alligator. Gen Comp Endocrinol.

[b27-ehp0114-001024] Kim E-Y, Hahn ME (2002). cDNA cloning and characterization of an aryl hydrocarbon receptor from
the harbour seal (*Phoca vitulina*): a biomarker of dioxin susceptivity?. Aquat Toxicol.

[b28-ehp0114-001024] Legler J, Brouwer A (2003). Are brominated flame retardants endocrine disruptors?. Environ Int.

[b29-ehp0114-001024] Levin MJ, De Guise S, Ross PS (2005). Association betwen lymphocyte proliferation and polychlorinated biphenyls
in free-ranging harbor seal (*Phoca vitulina*) pups from British Columbia, Canada. Environ Toxicol Chem.

[b30-ehp0114-001024] Mellish JE, Iverson SJ, Bowen WD, Hammill MO (1999). Fat transfer and energetics during lactation in the hooded seal: the roles
of tissue lipoprotein lipase in milk fat secretion and pup blubber
deposition. J Comp Physiol B.

[b31-ehp0114-001024] Miller KA, Assunção MGL, Dangerfield NJ, Bandiera SM, Ross PS (2005). Assessment of cytochrome P450 1A in harbour seals (*Phoca vitulina*) using a minimally-invasive biopsy approach. Mar Environ Res.

[b32-ehp0114-001024] Mos L, Ross PS (2002). Vitamin A physiology in the precocious harbour seal (*Phoca vitulina*): a tissue-based biomarker approach. Can J Zool.

[b33-ehp0114-001024] National Center for Biotechnology Information 2006. Basic Local Alignment Search Tool (BLAST). Available: http://www.ncbi.nlm.nih.gov/BLAST [accessed 27 April 2006].

[b34-ehp0114-001024] NOAA (National Oceanic and Atmospheric Administration) 1997. National Marine Fisheries Service. The Marine Mammal Protection Act of 1972 (as Amended through 1997). Available: http://www.nmfs.noaa.gov/pr/laws/MMPA/mmpatext/mmpaall.pdf [accessed 27 April 2006].

[b35-ehp0114-001024] Pelletier C, Imbeault P, Tremblay A (2003). Energy balance and pollution by organochlorines and polychlorinated biphenyls. Obes Rev.

[b36-ehp0114-001024] Reijnders PJH (1986). Reproductive failure in common seals feeding on fish from polluted coastal
waters. Nature.

[b37-ehp0114-001024] Rolland RM (2000). A review of chemically-induced alterations in thyroid and vitamin A status
from field studies of wildlife and fish. J Wildl Dis.

[b38-ehp0114-001024] Ross PS (2000). Marine mammals as sentinels in ecological risk assessment. HERA.

[b39-ehp0114-001024] Ross PS, Ellis GM, Ikonomou MG, Barrett-Lennard LG, Addison RF (2000). High PCB concentrations in free-ranging Pacific killer whales, *Orcinus orca*: effects of age, sex and dietary preference. Mar Pollut Bull.

[b40-ehp0114-001024] Ross PS, Jeffries SJ, Yunker MB, Addison RF, Ikonomou MG, Calambokidis J (2004). Harbour seals (*Phoca vitulina*) in British Columbia, Canada, and Washington State, USA, reveal a combination
of local and global polychlorinated biphenyl, dioxin, and furan
signals. Environ Toxicol Chem.

[b41-ehp0114-001024] Schumacher U, Zahler S, Horny HP, Heidemann G, Skírnisson K, Welsch U (1993). Histological investigations on the thyroid glands of marine mammals (*Phoca vitulina, Phocoena phocoena*) and the possible implications of marine pollution. J Wildl Dis.

[b42-ehp0114-001024] Shi YB, Liang VC (1994). Cloning and characterization of the ribosomal protein *L8* gene from *Xenopus laevis*. Biochem Biophys Acta.

[b43-ehp0114-001024] Simms W, Jeffries SJ, Ikonomou MG, Ross PS (2000). Contaminant-related disruption of vitamin A dynamics in free-ranging harbor
seal (*Phoca vitulina*) pups from British Columbia, Canada and Washington State, USA. Environ Toxicol Chem.

[b44-ehp0114-001024] Simms W, Ross PS (2000). Developmental changes in circulatory vitamin A (retinol) and its transport
proteins in free-ranging harbour seal (*Phoca vitulina*) pups. Can J Zool.

[b45-ehp0114-001024] Skaare JU, Bernhoft A, Wiig Ø, Norum KR, Haug E, Eide DM (2001). Relationships between plasma levels of organochlorines, retinol and thyroid
hormones from polar bears (*Ursus maritimus*) at Svalbard. J Toxicol Environ Health.

[b46-ehp0114-001024] Smith HO (1980). Recovery of DNA from gels. Methods Enzymol.

[b47-ehp0114-001024] Sonne C, Dietz R, Born EW, Riget FF, Kirkegaard M, Hyldstrup L (2004). Is bone mineral composition disrupted by organochlorines in East Greenland
polar bears (*Ursus martimus*)?. Environ Health Perspect.

[b48-ehp0114-001024] State of Washington Office of Financial Management 2000. Population Density 2000. Available: http://www.ofm.wa.gov/popden/colormap.asp [accessed 27 April 2006].

[b49-ehp0114-001024] Statistics Canada 2001. Population Distribution. Available: http://geodepot.statcan.ca/Diss/Maps/ThematicMaps/pop_dist_e.cfm [accessed 27 April 2006].

[b50-ehp0114-001024] Tanabe S, Watanabe S, Kan H, Tatsukawa R (1988). Capacity and mode of PCB metabolism in small cetaceans. Mar Mamm Sci.

[b51-ehp0114-001024] Van den Berg M, Birnbaum L, Bosveld ATC, Brunstrom B, Cook P, Feeley M (1998). Toxic equivalency factors (TEFs) for PCBs, PCDDs, PCDFs for humans and
wildlife. Environ Health Perspect.

[b52-ehp0114-001024] Veldhoen N, Helbing CC (2001). Detection of environmental endocrine-disruptor effects on gene expression
in live *Rana catesbeiana* tadpoles using a tail fin biopsy technique. Environ Toxicol Chem.

[b53-ehp0114-001024] Viguerie N, Millet L, Avizou S, Vidal H, Larrouy D, Langin D (2002). Regulation of human adipocyte gene expression by thyroid hormone. J Clin Endocr Metab.

[b54-ehp0114-001024] Wu Y, Koenig RJ (2000). Gene regulation by thyroid hormone. Trends Endcrinol Metab.

[b55-ehp0114-001024] Yen PM (2001). Physiological and molecular basis of thyroid hormone action. Physiol Rev.

[b56-ehp0114-001024] Zoeller RT (2005). Environmental chemicals as thyroid hormone analogues: new studies indicate
that thyroid hormone receptors are targets of industrial chemicals?. Mol Cell Endocrinol.

[b57-ehp0114-001024] Zoeller RT, Dowling ALS, Vas AA (2000). Developmental exposure to polychlorinated biphenyls exerts thyroid hormone-like
effects on the expression of RC3/neurogranin and myelin basic
protein messenger ribonucleic acid in the developing rat brain. Endocrinology.

